# Preclinical Evaluation of Human Donor-Derived Micronized Bone Marrow Stroma/Parenchyma Versus Bone Marrow Aspirate Concentrate in a Rat Model of Post-Traumatic Knee Osteoarthritis

**DOI:** 10.3390/cells15141249

**Published:** 2026-07-10

**Authors:** Haruki Nishimura, Zuokui Xiao, Jacob Singer, Xueqin Gao, William Sealy Hambright, Ryan Dregalla, Christopher T. Donner, Lucanus S. Koldewyn, Edward Jeffrey Donner, Johnny Huard

**Affiliations:** 1 The Steadman Philippon Research Institute, Vail, CO 81657, USA; hnishimura2468@outlook.com (H.N.); zxiao@sprivail.org (Z.X.); jacobsinger1@gmail.com (J.S.); xgao@sprivail.org (X.G.); 2 BMAX Medical LLC, Johnstown, CO 80534, USA; sealyhambright@yahoo.com (W.S.H.); rdregalla@dremedtech.com (R.D.); chrisdonner@bmaxmedical.com (C.T.D.); luc@colospine.com (L.S.K.); 3 Colorado Spine Institute, Johnstown, CO 80534, USA; 4 Elite Regenerative Stem Cell Specialists, Johnstown, CO 80534, USA; 5 Dregalla Medical Technologies LLC, Fort Collins, CO 80525, USA

**Keywords:** mesenchymal stromal cells, bone marrow niche, extracellular matrix, osteoarthritis, orthobiologics

## Abstract

**Highlights:**

**What are the main findings?**

- ECM-retaining micronized bone marrow (BMAX™) improved pain-related behavior and reduced joint swelling compared with conventional BMAC in a rat DMM osteoarthritis model.
- BMAX™ demonstrated improved cartilage regeneration potential, with significantly lower OARSI scores at 8 weeks after treatment compared to BMAC.

**What are the implications of the main findings?**

- Preservation of native bone marrow extracellular matrix may enhance the therapeutic efficacy of bone marrow–derived cell therapies for osteoarthritis.
- Maintaining the mesenchymal stromal/stem cell niche could represent an important strategy for optimizing regenerative medicine approaches.

**Abstract:**

Bone marrow aspirate concentrate (BMAC) is widely used as a source of mesenchymal stromal/stem cells (MSCs) for musculoskeletal regeneration; however, BMAC lacks essential bone marrow extracellular matrix (ECM) components, a critical component of the stem cell niche that regulates MSC survival, paracrine signaling, and regenerative capacity. We previously demonstrated that an ECM-retaining micronized bone marrow product (BMAX™) preserves pro-regenerative MSC phenotypes in vitro. Human bone marrow from a single donor was processed into conventional BMAC or BMAX™. Post-traumatic osteoarthritis was induced in immunodeficient rats using destabilization of the medial meniscus (DMM). Four weeks after surgery, animals were randomly assigned to receive intra-articular injections of BMAX™, BMAC, or phosphate-buffered saline (*n* = 10–12 in each group). Pain-related behavior (*n* = 5–6/group), histological assessment (*n* = 3–6/group), and micro-computed tomography (*n* = 4–6/group) were evaluated for up to 8 weeks after treatment. At 4 weeks after treatment, BMAC significantly increased the paw withdrawal threshold compared with PBS (*p* = 0.0068), whereas BMAX™ significantly reduced knee joint swelling compared with both PBS (*p* = 0.0235) and BMAC (*p* = 0.0039), and BMAX™ significantly improved knee bend scores compared with PBS (*p* = 0.0011). Neither treatment significantly improved OARSI histological scores at this time point. At 8 weeks after treatment, BMAX™ significantly increased the paw withdrawal threshold compared with PBS (*p* = 0.0305), whereas BMAC showed a non-significant trend (*p* = 0.0517); both treatments significantly reduced knee bend scores compared with PBS (*p* = 0.0027 and *p* = 0.0255), and BMAX™ demonstrated significantly lower knee bend scores than BMAC (*p* = 0.0090). BMAX™ significantly reduced knee swelling compared with PBS (*p* = 0.0196). Histologically, BMAX™ significantly improved OARSI scores in both the femoral condyle and tibial plateau compared with PBS (*p* = 0.0020 and *p* = 0.0003, respectively), whereas BMAC significantly improved only tibial plateau OARSI scores (*p* = 0.0014). Furthermore, BMAX™ demonstrated significantly lower femoral condyle OARSI scores than BMAC (*p* = 0.0243). Micro-computed tomography revealed that both BMAX™ and BMAC significantly reduced medial subchondral trabecular separation compared with PBS (*p* = 0.0340 and *p* = 0.0426, respectively), whereas no significant differences were observed between the two treatment groups for other bone structural parameters. In conclusion, preservation of the native bone marrow ECM was associated with improved functional outcomes and greater cartilage regeneration compared with conventional BMAC in this preclinical rat model of post-traumatic osteoarthritis. These findings support the concept that maintaining the native stem cell niche may enhance the therapeutic potential of bone marrow-derived cell therapies for osteoarthritis.

## 1. Introduction

Mesenchymal stem cells, or medicinal signaling cells (MSCs), are multipotent progenitor cells capable of differentiating into adipocytes, osteoblasts, and chondrocytes. In addition to their differentiation potential, MSCs exert significant paracrine effects through the release of cytokines and growth factors that support anti-inflammatory responses and tissue regeneration. Bone marrow aspirate concentrate (BMAC) has gained popularity as an autologous MSC source for a wide range of regenerative medicine applications, including knee osteoarthritis (OA) [1,2,3,4,5,6,7].

The bone marrow niche itself includes both cellular and structural elements that critically influence the behavior of resident stem cells, including MSCs and hematopoietic stem cells (HSCs). It is known that components of the microenvironment, especially extracellular matrix (ECM) components, modulate MSC proliferation, self-renewal, and lineage commitment while shaping a pro-regenerative secretome profile [8,9]. The ECM within the marrow stroma is rich in proteoglycans, glycosaminoglycans (GAGs), and various types of collagens supporting cell adhesion, migration, and intercellular signaling that are fundamental for both hematopoiesis and osteogenesis [10,11]. Recent spatial mapping of the human marrow niche showed that MSC subtypes are non-randomly distributed relative to the bone surface, with osteogenic and ISCT-defined fibroblastic (CD73/CD90/CD105^+^) MSCs concentrated at the endosteal, bone-adjacent niche and adipogenic MSCs residing more centrally [12]. Notably, that work observed that marrow aspiration “fails to capture cells that are tightly adhered to the bone surface” and biases recovered MSCs toward an adipocytic phenotype. Because BMAX™ is obtained as subcortical bone-core stroma/parenchyma with minimal agitation rather than by high-volume aspiration, it is plausible that BMAX™ and BMAC contain at least partially distinct MSC subpopulations defined by their native niche—a possibility consistent with the enrichment of CD146^+^ perivascular (pericyte) cells in BMAX™-derived cultures. We did not directly resolve MSC subpopulation identity in the two preparations, and such phenotyping is an important objective for future study.

The primary goal of BMAC processing is to achieve a concentrated dose of MSCs in a smaller injection volume to be delivered to the site of injury or repair. However, MSCs are inherently rare in BMA, comprising approximately 0.001% to 0.01% of total nucleated cells [13,14]. Bone marrow aspirate can be harvested from several anatomical sites, although the posterior iliac crest is generally preferred because it is readily accessible and typically provides higher yields of MSCs along with richer concentrations of cytokines and growth factors [15]. However, studies have shown that drawing such large cumulative volumes (generally > 120 mL) introduces dilutive peripheral blood, resulting in diminishing cellular returns [13,16,17] and potential deleterious effects known to occur with MSCs exposed to red blood cells and their releasates [18,19].

We previously reported a human bone marrow harvesting and processing technique for subcortical bone core samples that retain key ECM components of native stroma with minimal mechanical agitation in an intraoperative window of time [9]. The resulting micronized bone marrow product (BMAX™) contains MSCs in relatively higher abundance compared to BMAC with unique functional phenotypes in vitro [9]. BMAX™ derived MSCs exhibited a unique and more pro-regenerative secretome with increased adherence, proliferation, and relatively more stromal-derived MSCs versus traditional BMAC centrifugation-based preparations.

Fundamentally, conventional BMAC is derived from aspirated marrow fluid, which is diluted by peripheral blood and biased toward adipocytic MSCs, largely missing the bone-adherent, clonogenic ISCT-defined MSCs [12], whereas BMAX™ is harvested as intact marrow tissue (subcortical bone cores) that preserves the native stromal ECM and stem-cell niche. Whether this tissue-preserving approach yields greater therapeutic efficacy in vivo, and specifically superiority over conventional BMAC in osteoarthritis, has not been directly tested. In the present study, building on our prior in vitro characterization of BMAX™ [9], we evaluated its therapeutic effects in a rat destabilization of the medial meniscus (DMM) model of post-traumatic osteoarthritis and compared its efficacy with conventional BMAC. We hypothesized that preservation of the native bone marrow ECM would enhance the regenerative potential of bone marrow–derived cell therapy. Our findings demonstrated that BMAX™ improved pain-related behavior, reduced joint swelling, and resulted in superior cartilage preservation/regeneration compared with conventional BMAC, highlighting the importance of maintaining the native stem cell microenvironment in regenerative strategies for OA.

## 2. Materials and Methods

### 2.1. Human Bone Marrow Collection and Preparation

Bone marrow subcortical bone cores and aspirate were obtained from a single consented 50-year-old female donor (height 5 ft 6 in, weight 121 lb, BMI 19.5) with Ehlers–Danlos syndrome, in conjunction with her own clinical bone-marrow treatment procedure (WCG-IRB# 20230413). She was selected from among several prospective donors specifically because she carried among the highest burden of senescent cells (C12FDG^+^/CD87^+^) on pre-procedural screening, representing a conservative, worst-case regenerative profile. A single donor was used by design so that BMAX™ and BMAC were prepared from the same marrow, enabling a controlled, donor-matched comparison of the two processing methods free of inter-donor variability. Bone marrow aspirate and subcortical bone cores were harvested from the iliac crest on two occasions; on each occasion the harvest was bilateral, with one side used for the donor’s own clinical treatment and the contralateral iliac crest used to obtain the material for this study. Material from the two sessions provided donor-matched preparations for two rat cohorts; pooling these cohorts accounts for the differences in group sizes across endpoints. Aspirate was anticoagulated with preservative-free heparin at a final concentration of 10 U/mL. The BMAC sample was prepared from 50 mL of bone marrow aspirate by two-step centrifugation as described [4]. For BMAX™, bone cores were harvested with an 11-gauge Jamshidi needle and processed using the BMAX™ device (BMAX Medical LLC, Johnstown, CO, USA) as previously described [9]. Briefly, the core was pressed into the device, which was washed with heparin and contained 1 mL of saline; upon completion of BMAC processing, approximately 2 mL of bone marrow-derived plasma was added for a total volume of 3 mL, and the device underwent two automated cycles according to the manufacturer’s preprogrammed processing parameters, which are proprietary and not disclosed, after which the BMAX™ product was withdrawn into a 3 mL syringe. BMAC and BMAX™ were processed immediately after harvest, without interim storage, and were transported to the animal facility (approximately 30 min away) for administration; all material was administered fresh, without cryopreservation, and the treated animals were dedicated study arms that received no other interventions. Absolute total nucleated cell and MSC concentrations of the fresh injectate were not enumerated; the final intra-articular injectate volume was 100 µL for all groups.

### 2.2. BMAX Processing

**Figure 1:**
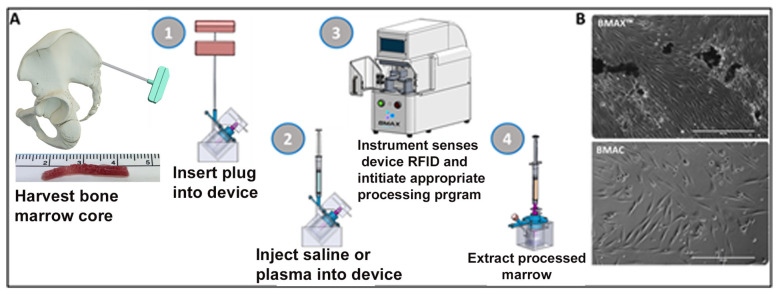
BMAX™ processing methodology. (**A**) Schematic illustration of the mechanical processing workflow used to generate an injectable, ECM-retaining micronized bone marrow preparation. Bone cores harvested from the iliac crest are mechanically processed to preserve native extracellular matrix components within a micronized marrow product suitable for intra-articular delivery. (**B**) Morphology of BMAX and BMAC-derived MSCs.

Mechanical processing of human BC using the BMAX™ device (Figure 1) was previously shown to retain demineralized GAG-rich ECM in culture conditions resulting in differential MSC phenotypes, including proliferative capacity and secretome, compared to donor-matched BMAC [9]. To verify similar MSC characteristics for the donor used here, extracts from both BMAC and BMAX™ were analyzed by flow cytometry to calculate relative MSC yield, then plated to evaluate colony-forming units (CFUs).

### 2.3. CFU-f Assays

BMAX™ and BMAC samples were cultivated as described [19,20]. Colony-forming unit–fibroblast (CFU-F) assays were performed to quantify clonogenic mesenchymal stromal cell (MSC) precursors obtained from bone marrow samples. Briefly, bone-marrow mononuclear cells were plated at 1 × 10^6^ cells per 35 mm dish in DMEM supplemented with 20% FBS and antibiotics, and cultured at 37 °C, 5% CO_2_ with medium changes every 3 days. After 14 days (P0), adherent cultures were fixed (4% formaldehyde), stained (0.05% crystal violet), and colonies consisting of ≥50 fibroblast-like cells were counted and reported as CFU-F per mL marrow (or per 10^5^ BM-MNCs), and overall colony growth was further quantified using a colorimetric method with a spectrophotometer.

### 2.4. Flow Cytometry

MSCs obtained from both BMAX™ and BMAC cultures were identified via flow cytometry following the International Society for Cell & Gene Therapy (ISCT) minimal criteria for multipotency [21] (minimum 10,000 cells analyzed) as previously described CD34^−^, CD4^5−^, CD11b^−^, CD19^−^, HLA-DR^−^, CD73^+^, CD90^+^, CD105^+^ (BD Biosciences, San Jose, CA, USA. Stem Flow Cat.# 562245) [9]. Subcultures were continued through passage 2 and analyzed at passages 0 (from primary culture), 1 and 2 for pericyte phenotype via CD45^−^ (PerCPCy5.5-conjugated)/CD146^+^ (FITC-conjugated) labeling (Biolegend, San Diego, CA, USA. Cat.# 304028, 361012, respectively). For each panel, regions for the cultured cell population were established based on FSC/SSC plots. Antibody isotype controls were used for each fluorescent channel to determine negative fluorescence (no signal) based on the respective channel’s histogram on a log scale. The panel following ISCT criteria (BD BioSciences) used isotype controls (provided by the manufacturer) to establish the appropriate positive/negative gates in each channel. The cell population that were negative in the PE-channel for the WBC antibody cocktail (CD34^−^, CD45^−^, CD11b^−^, CD19^−^, HLA-DR^−^) were used as the starting criteria and backgated in a serial fashion for CD73^+^ (WBC^−^, CD73^+^), to CD105^+^ (WBC^−^, CD73^+^, CD105^+^) to CD90^+^ (WBC^−^, CD73^+^, CD105^+^, CD90^+^) (Supplementary Figure S1) Similarly, the appropriate antibody isotypes were used to set initial gates for the CD45 and CD146 panel. The CD146^+^ population in the respective channel was backgated to the CD45^−^ population to determine the percent of CD45^−^/CD146^+^ cells (Supplementary Figure S1).

### 2.5. Animal Study Design

All animal housing and procedures were performed at the Colorado State University animal facility under an approved IACUC protocol (Protocol #1322). Adult female (12 weeks of age, weighed approximately 250–300 g) RNU Nude rats (Charles River Laboratories, Crl: NIH-Foxn1rnu) were randomly assigned to 3 treatment groups (10–12 rats per group): BMAX™, BMAC, and PBS controls. No formal a priori sample size calculation was performed. Group sizes were determined based on prior studies using the DMM model and feasibility considerations. Contralateral knees were also examined after sacrifice as there were no treatment controls. Treatments were administered intra-articularly under anesthesia with Sevoflurane at 4 weeks after induction of OA with DMM surgery. Five rats per group were evaluated for pain behavior prior to DMM surgery, 4 weeks after DMM, and at 4 and 8 weeks following injection. Swelling of the affected joint was also evaluated at 4 and 8 weeks after injection. 5–6 rats per group were allocated for histological and micro-CT analyses at 4 and 8 weeks after injection. Owing to technical issues during image acquisition or tissue processing, the final sample size differed slightly for some analyses, as reported in the corresponding tables and figure legend. The primary outcome measures were knee-bend pain score and OARSI histological score at 8 weeks post-injection. Animals were eligible for inclusion if they successfully underwent the DMM procedure and survived to the designated experimental endpoint. Prespecified exclusion criteria included major surgical complications (e.g., postoperative infection or perioperative mortality) that would preclude completion of the study protocol. No animals met these exclusion criteria, and no animals or data points were excluded from the final analyses.

Four weeks after DMM surgery, animals were randomly assigned to the treatment groups immediately prior to intra-articular injection. Because treatment allocation occurred after completion of the surgical procedure, surgeon blinding during DMM surgery was not applicable. Due to the nature of the intervention, the investigator administering the intra-articular injections was aware of the treatment allocation. However, all behavioral, histological, and micro-CT outcome assessments were performed by investigators blinded to treatment allocation.

### 2.6. Anesthesia and Euthanasia

All surgical procedures were performed under nose cone inhalational anesthesia using Isoflurane (2–3% in oxygen) delivered via a precision vaporizer to maintain a surgical plane of anesthesia throughout the procedure. Animals were monitored for depth of anesthesia by assessment of respiratory rate and pedal withdrawal reflex.

At the experimental endpoint (4 or 8 weeks post-injection), euthanasia was performed by carbon dioxide (CO_2_) inhalation using a gradual fill method in accordance with institutional animal care guidelines and the American Veterinary Medical Association (AVMA) Guidelines for the Euthanasia of Animals. Death was confirmed by cessation of respiration and absence of reflexes.

Following confirmation of death, terminal cardiac puncture was performed for blood collection for potential future analyses. No blood-derived data were included in the present study.

### 2.7. Post-Traumatic Knee Osteoarthritis Model

The DMM model was selected as a well-established and reproducible model of post-traumatic osteoarthritis (PTOA) to evaluate cartilage degeneration and pain-related behavior. DMM surgeries were performed as described [22]. In short, a medial capsular incision was made on the right knee to expose the joint after anesthesia was induced with Sevoflurane. The extensor muscles of the knee were carefully retracted laterally, ensuring the patellar tendon remained intact. The medial meniscotibial ligament was then severed, allowing the medial meniscus to be shifted medially. Following this procedure, the extensor muscles were repositioned, the capsule was sutured, and the skin was closed. All surgical procedures were performed by the same investigator to minimize procedural variability.

### 2.8. Live Animal Pain and Knee Joint Swelling Assessment

Nociception was behaviorally evaluated in all animals by both the Von Frey and knee-bend scoring methods as described [23,24]. Mechanical sensitivity was assessed using a calibrated electronic von Frey device (IITC, Woodland Hills, CA, USA). Following a period of habituation, the von Frey probe was applied perpendicularly to the hind paw with constant force (no ramping) and maintained for 6 to 8 s. The withdrawal threshold was measured three times for each animal, with approximately 10 min intervals between trials, and the mean value was used for analysis. The Knee-Bend test involves assessing the animal’s behavioral responses, such as vocalizations (squeaks) and physical resistance, during five cycles each of passive knee flexion and extension. Reactions are scored using a defined scale: a score of 0 indicates no response during any movement, and the highest score corresponds to squeaking in response to mid-range movements. The total Knee-Bend score, with a maximum of 20, serves as a behavioral index of joint-related nociception triggered by movement. To eliminate bias, behavioral testing was carried out by an investigator blinded to the experimental grouping of the animals. To assess joint swelling, the diameters of the right and left knee joints were measured using digital calipers at 4 and 8 weeks after the injection. The knee joint diameter was defined as the distance between the lateral and medial collateral ligament regions [25]. The differences in knee diameter between right and left knee joints for each animal were calculated, and the results were averaged for each group at each evaluation time. These assessments were conducted in a consistent order and at similar times of day.

### 2.9. Micro-CT Analysis

Micro-CT was performed after knee tissue harvest and formalin fixation. Knee tissues were scanned with Viva CT 80 (Scanco Medical LLC, Brüttisellen, Switzerland) using a Voxel size of 10 µm, 70 kvP and 114 µA. After the acquisition of 2D images, the 3D overview was analyzed first with consistent dimensions to obtain an overview of the entire knee to reveal the general morphology of the proximal tibia. Subsequently, medial and lateral proximal tibia subchondral bone were defined by contouring the view of interest right below the articular cartilage for quantification of microarchitecture. 3D bone microarchitecture quantification was analyzed using Gauss sigma = 0.8, Gauss support = 1 and a threshold of 220 in the subchondral bone of the affected knee joint as described [26]. Bone volume and density and other parameters were generated automatically by Micro-CT software (Version 6.1). The nomenclature of bone microarchitecture followed the guidelines of the American Society of Bone and Mineral Research [27].

### 2.10. Histology and Immunohistochemistry of Articular Cartilage

After micro-CT scanning, bone tissues were decalcified using Immunocal™ Decalcifier (Stat Lab, McKinney, TX, USA) for 4 weeks and then processed and paraffin-embedded. Sections were cut at the sagittal plane from the medial side at 5 µm thickness. Alcian blue and safranin-O staining was performed as described [26]. Microscopic images were taken at 40× and 100× for the entire femur condyle and tibia plateau cartilage using a NIKON CI microscope (Nikon, Melville, NY, USA). Histology score was evaluated using OARSI score criteria as previously described [28]. Immunohistochemistry of collagen type 2 (COL2) was performed according to previously published work [29].

### 2.11. Statistical Analysis

Results are presented as mean ± SD. Data were analyzed in GraphPad Prism version 10.1.2. Data distribution was assessed for normality prior to parametric testing. When assumptions of normality were not met, non-parametric tests were applied. For pain testing, which occurred in the same animals over multiple timepoints, two-way repeated-measures ANOVA was used. One-way ANOVA, followed by Tukey’s post hoc test, was applied when three or more groups were analyzed at the same timepoints. For comparisons involving only two groups, the Mann–Whitney U test was applied. A *p*-value of less than 0.05 was considered statistically significant.

A post hoc power analysis was performed using G*Power (version 3.1) based on the observed difference in the 12-week knee bend score between the BMAC and BMAX groups, which represented the primary functional outcome supporting the comparison of treatment efficacy. Achieved power was calculated using a two-tailed independent-samples *t*-test with an α level of 0.05.

This study was reported in accordance with the ARRIVE guidelines 2.0.

## 3. Results

### 3.1. BMAX Cells Favor Pericyte Expansion During Culture

**Figure 2:**
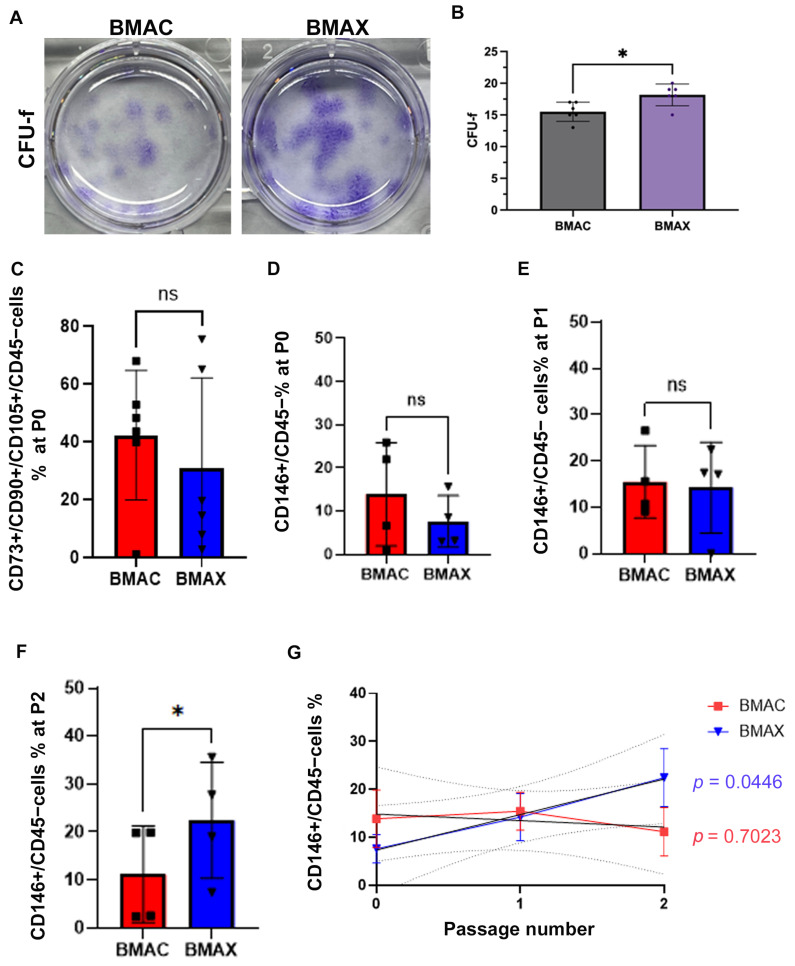
Characterization of micronized bone marrow cells. (**A**,**B**) Representative colony-forming unit–fibroblast (CFU-F) assay images and quantitative analysis of CFU-F formation from BMAC and BMAX after 14 days in culture. (**C**) Flow cytometric analysis of mesenchymal stromal/stem cell populations CD73^+^CD90^+^/CD105^+^/CD45^−^ adhering cells % according to ISCT criteria in BMAC and BMAX samples. (**D**–**F**) CD146^+^/CD45^−^ pericyte % adhering cells at different passages. (**G**) CD146^+^/CD45^−^ pericyte % across passages 0–2 (BMAX *p* = 0.0446). Data are presented as mean ± SD. Significant difference: * *p* < 0.05. ns: not significant.

We further performed a CFU-F assay to determine the cell expansion of BMAX- and BMAC-derived adhering cells. We found adhering cells from BMAX formed more colonies in 14 days (Figure 2A,B). MSCs marker profiling of adhering cells at P0 demonstrated no significant difference in MSC % in the BMAX group compared to the BMAC group (Figure 2C). However, the percentage of CD146^+^CD45^−^ cells in BMAX-derived adhering cells increased over passages and significantly increased at P2 compared to BMAC-derived adhering cells (Figure 2D–G).

### 3.2. Experimental Design and In Vivo Evaluation Using Knee Osteoarthritis Model Rats

**Figure 3:**
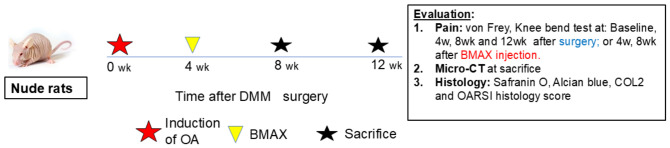
Experimental design and study timeline. Overview of the experimental design. Post-traumatic osteoarthritis was induced using the destabilization of the medial meniscus (DMM) model. Intra-articular injections of ECM-retaining micronized bone marrow, BMAC, or vehicle control were administered 4 weeks after surgery. Behavioral assessments, joint swelling measurements, histological analyses, and micro-computed tomography were performed at the indicated time points.

To evaluate therapeutic efficacy in vivo, DMM surgery was performed in immunodeficient rats to permit administration of human bone marrow–derived preparations. Animals received intra-articular injections of ECM-retaining micronized bone marrow, BMAC, or vehicle control (PBS) four weeks after DMM surgery (Figure 3). Equal injection volumes (100 µL) were used across all treatment groups. No signs of infection or adverse local reactions were observed in any group throughout the study period.

### 3.3. ECM-Retaining Micronized Bone Marrow Improves Pain-Related Behaviors and Reduces Joint Swelling After DMM Surgery

**Figure 4:**
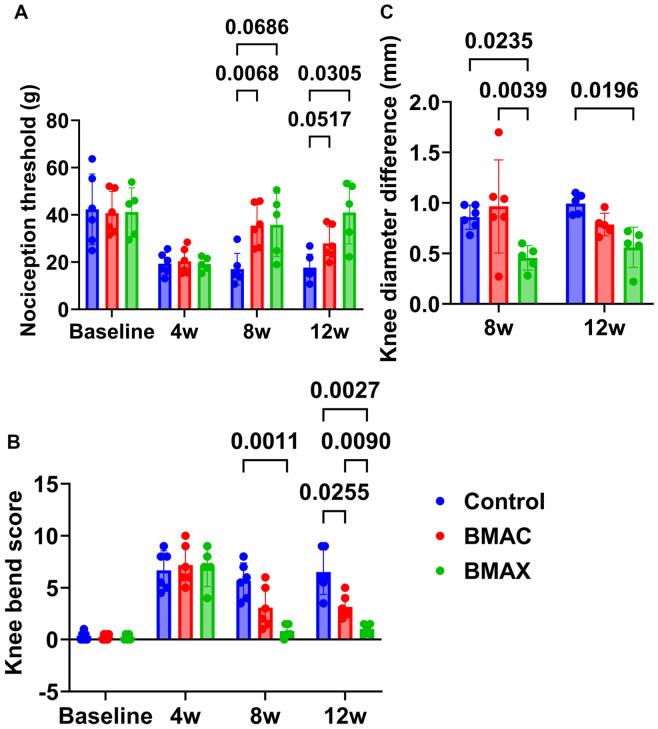
Pain-related behaviors and joint swelling after treatment. (**A**) Mechanical sensitivity assessed by von Frey testing at baseline, after DMM surgery, and at 4 and 8 weeks following treatment. (**B**) Movement-evoked pain assessed using the knee-bend test at corresponding time points. (**C**) Joint swelling quantified as differences in knee diameter between operated and contralateral limbs at 4 and 8 weeks post-treatment. Data are presented as mean ± SD. Each dot represents one animal, *n* = 6 for the PBS group, *n* = 5 for the BMAC and BMAX group. Post hoc multiple comparison exact *p* values are indicated between group bars.

Pain-related behaviors were assessed using both von Frey mechanical sensitivity testing and the knee-bend assay at baseline, after DMM surgery, and at 4 and 8 weeks following treatment. DMM surgery resulted in increased nociceptive responses in all animals prior to treatment. In the von Frey assay, BMAC significantly increased the nociception threshold versus PBS at 4 weeks post-injection (*p* = 0.0068), with BMAX™ showing a comparable trend (*p* = 0.0686); by 8 weeks, BMAX™ reached significance versus PBS (*p* = 0.0305) while BMAC showed a trend (*p* = 0.0517), and BMAX™ and BMAC did not differ significantly from each other at either time point (Table 1, Figure 4A). In the knee-bend assay, BMAX™ significantly reduced movement-evoked pain versus PBS at both 4 and 8 weeks post-injection (*p* = 0.0011 and *p* = 0.0027), as did BMAC at 8 weeks (*p* = 0.0255); BMAX™ scored significantly lower than BMAC at 8 weeks post-injection (*p* = 0.0090), with no significant difference at 4 weeks (Table 2, Figure 4B). A post hoc power analysis based on the 12-week knee bend score demonstrated an achieved power of 0.940 (Cohen’s d = 2.40) for the comparison between the BMAC and BMAX groups.

Joint swelling was evaluated by measuring differences in knee diameter between operated and contralateral limbs. Animals receiving ECM-retaining micronized bone marrow demonstrated significantly reduced joint swelling compared with BMAC at 4 weeks and compared with PBS at both 4 and 8 weeks post-injection (Table 3, Figure 4C). No significant differences in swelling were observed between BMAC and PBS groups at these time points.

### 3.4. ECM-Retaining Micronized Bone Marrow Attenuates Cartilage Degeneration in the Late Stage of Knee OA

**Figure 5:**
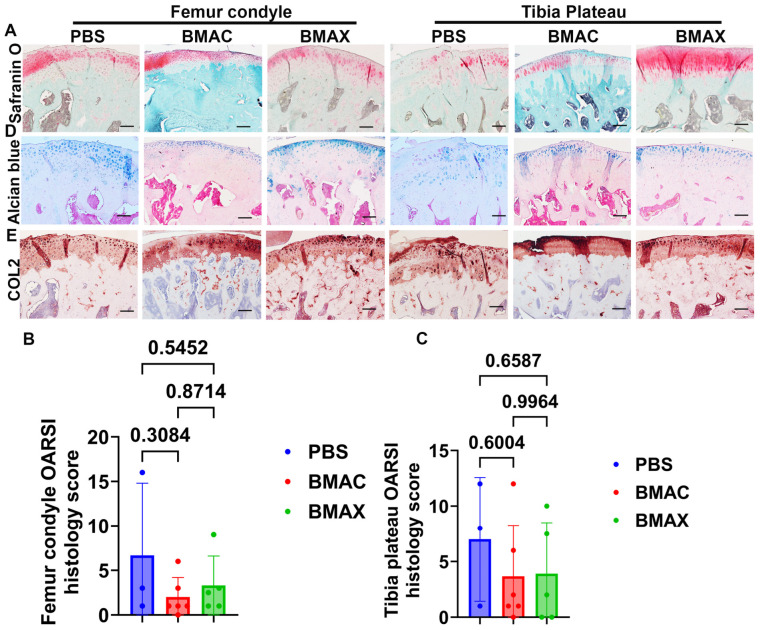
Cartilage histology after 4 weeks of treatment. (**A**) Representative Safranin O–stained sections of femoral condyle and tibial plateau cartilage at 4 weeks post-treatment. (**B**,**C**) Quantitative Osteoarthritis Research Society International (OARSI) scores for femoral condyle and tibial plateau cartilage. (**D**) Representative Alcian blue–stained sections demonstrating proteoglycan distribution. (**E**) Immunohistochemical staining for type II collagen in femoral condyle and tibial plateau cartilage. Data are presented as mean ± SD. Exact *p* values are indicated between group bars.

Histological evaluation of articular cartilage was performed at 4 and 8 weeks following treatment using Safranin O and Alcian blue staining, with cartilage degeneration quantified using OARSI scoring criteria. At 4 weeks post-treatment, no significant differences in OARSI scores were observed among treatment groups (Table 4 and Figure 5A–C).

**Figure 6:**
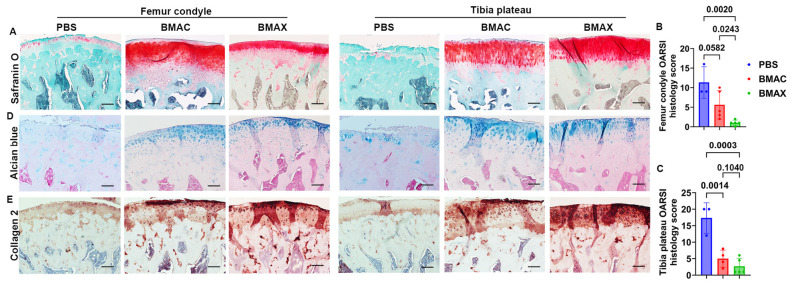
Cartilage histology after 8 weeks of treatment. (**A**) Representative Safranin O–stained sections of femoral condyle and tibial plateau cartilage at 8 weeks post-treatment. (**B**,**C**) Quantitative Osteoarthritis Research Society International (OARSI) scores for femoral condyle and tibial plateau cartilage. (**D**) Representative Alcian blue–stained sections demonstrating proteoglycan distribution. (**E**) Immunohistochemical staining for type II collagen in femoral condyle and tibial plateau cartilage. Data are presented as mean ± SD. Exact *p* values are indicated between group bars.

At 8 weeks post-treatment, PBS-treated joints exhibited marked loss of proteoglycan staining and structural cartilage damage in both femoral condyle and tibial plateau regions (Figure 6A). In contrast, joints treated with either ECM-retaining micronized bone marrow or BMAC demonstrated preservation of Safranin O and Alcian blue staining. Quantitative analysis revealed significantly improved OARSI scores in both treatment groups compared with PBS controls, with ECM-retaining micronized bone marrow showing significantly lower OARSI scores than BMAC in the femoral condyle region (Table 5 and Figure 6B,C).

Immunohistochemical analysis further demonstrated increased COL2 staining intensity in both treatment groups relative to PBS controls, with more uniform COL2 distribution observed in joints treated with ECM-retaining micronized bone marrow (Figure 6E).

### 3.5. Micro-Computed Tomography Reveals Modest Preservation of Subchondral Bone Architecture

**Figure 7:**
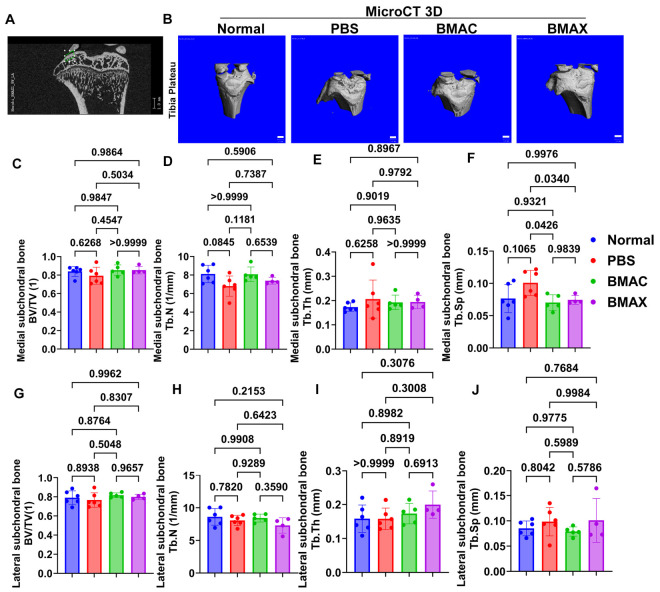
Micro-CT assessment of subchondral bone architecture after 8 weeks of treatment. (**A**). View of interest for analysis of subchondral bone. Green box indicates the area of analysis (view of interest). (**B**) Representative two-dimensional and three-dimensional micro-computed tomography images illustrating the region of interest in the medial proximal tibia following DMM surgery. (**C**–**F**) Quantitative analysis of medial subchondral bone parameters. (**G**–**J**) Quantitative analysis of lateral subchondral bone parameters. Data are presented as mean ± SD. Exact *p* values are indicated between group bars. Normal (*n* = 6), PBS (*n* = 6), BMAC (*n* = 5), BMAX™ (*n* = 4).

Micro-computed tomography analysis was performed to assess subchondral and trabecular bone architecture in the proximal tibia. The view of interest for the subchondral bone quantification is shown in Figure 7A. At 8 weeks after treatment, three-dimensional reconstructions confirmed successful induction of PTOA following DMM surgery, as evidenced by medial meniscal displacement across all groups (Figure 7B). On the medial side of the proximal tibia, compared with the uninjured contralateral limb (normal), PBS-treated joints demonstrated trends toward reduced trabecular number and increased trabecular separation in the medial subchondral region. Treatment with ECM-retaining micronized bone marrow or BMAC significantly attenuated Tb.Sp compared to the PBS group (*p* = 0.0340 and 0.0246, respectively); however, no statistically significant differences were observed among groups for bone volume fraction or trabecular thickness (Figure 7C–J). No significant differences were detected in lateral compartment subchondral trabecular bone parameters.

## 4. Discussion

BMAC is widely used to deliver MSCs to affected tissues with positive clinical outcomes for idiopathic and post-traumatic osteoarthritis [30,31]. However, emerging evidence suggests a lack of MSC persistence and regenerative attributes at the site of injury, which may be due to disrupted focal adhesion-linked pathways and signaling caused by the disruption of the MSC-ECM connection found in the bone marrow microenvironment [32,33]. A critical component of the marrow niche is the physical ECM micro-architecture, which contains proteoglycans, collagens, glycosaminoglycans, and a mixture of matricellular proteins [10,11]. The ECM functions as a reservoir for regenerative protein factors and remodeling proteases [34], providing a scaffold for cell–cell communication and receptor engagement that regulates stem cell localization and migration [11,35]. Rather than aspirating fluid, BMAX™ harvests subcortical bone cores, capturing MSCs from the bone-adherent compartment. Single-cell mapping of human marrow shows that bone marrow aspiration fails to capture cells tightly adhered to the bone surface and is biased toward adipocytic MSCs, whereas the ISCT-defined MSCs (CD73/CD90/CD105) with the highest clonogenic and proliferative capacity are largely absent from aspirate-derived samples [12]. Capturing this bone-associated MSC population, rather than a blood-diluted aspirate fraction, may therefore contribute to the greater adherence, proliferation, and clonogenicity of BMAX™-derived cells observed here and previously [9].

This study evaluated the ability of a novel processing technique to generate micronized ECM-rich bone marrow (BMAX™) to reduce PTOA progression in vivo using an established rat injury model. Interestingly, BMAX™ treatment was found to be just as effective as BMAC in reducing OA symptoms and even resulted in enhanced efficacy for certain outcomes. Processing limitations, variance in efficacy, and lack of compelling data directly supporting tissue restoration have all been reported for BMAC in preclinical and clinical studies. For example, the addition of autologous BMAC to juvenile allogeneic chondrocyte implantation improves patient-reported scores, yet radiographic imaging still reveals fibrocartilage [36,37] predominantly. Similarly, the combination of BMAC and PRP for large, full-thickness chondral defects was found to improve patient-reported symptoms, but the regenerated cartilage was still inferior to native hyaline cartilage [38]. These shortcomings could be related to the low proportion of reparative MSCs in BMAC per volume, being about 0.1–1.5% of total nucleated cells [14,39]. Micronized bone marrow akin to the BMAX™ preparations used here may circumvent these limitations through the retention of the physicochemical parameters of the native stroma microenvironment, improving the regenerative functionality of MSCs that is absent from BMAC preparations derived from marrow aspirate. The fact that BMAX™ treatment groups resulted in significantly better-preserved cartilage after injection may offer key insights into improved methods to mitigate such observed limitations with traditional BMAC preparations.

Current BMAC preparation techniques not only neglect the critical ECM structural components of the marrow stroma but often aspirate large marrow volumes that are significantly diluted with blood [4,40]. Previous studies investigating BMAC preparation protocols with different devices or techniques have routinely found the products to yield heterogeneous properties [13,40]. There is also significant variance in marrow aspirate draw volumes, which often exceed 120 cc (either unilateral or bilateral), which introduces significant amounts of diluting blood [13,17]. The BMAX™ method results in a smaller concentrated injectate volume, which mitigates the effects of diluting blood and associated erythrocytes, which our group and others have shown to negatively impact MSC properties and tissue repair ability [18,19]. Thus, the lack of a high erythrocyte count (hematocrit) may have led to the improved OARSI cartilage scores compared to BMAC. Additionally, this was somewhat supported by the micro-CT findings whereby subchondral bone microarchitecture was improved in BMAX™ treatment groups over BMAC in trabecular separation (Tb.Sp) scores.

The enhanced reduction in movement-evoked pain and knee swelling found in BMAX™ versus BMAC-treated rats may be explained by their differences in immunomodulatory secretome profiles and differences in leukocyte content of the two products. BMAX™ derived MSCs were previously found to exhibit enhanced responsiveness to immunomodulatory stimulation with TNF-α compared to BMAC [9]. TNF-α is a pro-inflammatory cytokine upregulated at injury sites that elicits paracrine signaling in MSCs, prompting an anti-inflammatory response important for inflammatory balance and resolution during healing [33,41]. In OA, TNF-α and IL-1β augment one another, promoting inflammation, cartilage degeneration, and pain [42,43]. IL-1 receptor antagonist (IRAP) downregulates IL-1β as a competitive antagonist and is an emergent target for novel OA treatments [42,43]. After TNF-α challenge in vitro, BMAX™ derived MSCs (non-culture-expanded) expressed IRAP, and when compared to donor-matched BMAC MSCs, BMAX™ MSCs responded to TNF-α exposure with significantly elevated levels of IL-6. Interestingly, IL-6 is known to elicit both anti-inflammatory and pro-inflammatory effects important for retaining MSC “stemness” or “plasticity” within the bone marrow niche [44,45]. The leukocyte chemoattractant chemokine IP-10 was also found to be markedly increased in BMAC-MSCs over 8 days in culture compared to the BMAX™ derived MSCs. Such an increase in IP-10 found in BMAC-MSCs is likely due to the vastly dominant leukocyte concentration found in BMAC (≥99.9% of cells). BMAX™ is a cell-tissue construct product with relatively lower TNC counts versus BMAC that lacks such enrichment in leukocytes [9]. These findings from our previous in vitro study suggest that differences in leukocyte content and immunomodulatory secretome profiles between BMAX™ and BMAC may contribute to the distinct therapeutic responses observed in the present study. However, inflammatory mediators such as IL-6 and IRAP were not directly evaluated in the current investigation. Therefore, any mechanistic contribution of these factors remains speculative and should be interpreted with caution. Future studies incorporating cytokine profiling and molecular analyses will be necessary to determine whether these pathways underlie the reduced joint inflammation and improved functional outcomes observed following BMAX™ treatment.

This study did have limitations. First, the number of animals was relatively small, which may have limited the statistical power to detect differences for some outcome measures, particularly those demonstrating only trends toward significance. Although significant differences in cartilage histology were observed at 8 weeks, OARSI scores at 4 weeks showed only favorable trends. Future studies with larger sample sizes may better define the magnitude and temporal progression of the therapeutic effects. Second, treatment was administered at a single time point (4 weeks after DMM surgery), and alternative intervention time points during OA progression were not evaluated. Future studies should investigate whether earlier or later administration further optimizes therapeutic efficacy. Third, only a single intra-articular injection was evaluated. Although this design enabled assessment of the therapeutic potential of BMAX™ under standardized experimental conditions, repeated administration may better reflect current clinical orthobiologic treatment strategies. Future studies should evaluate the effects of multiple injections, including optimal dosing intervals and treatment schedules, to determine whether repeated administration further enhances therapeutic efficacy. Fourth, both BMAC and ECM-retaining micronized bone marrow were prepared from a single human donor. Because MSC abundance, secretory profile, and extracellular matrix composition are known to vary according to donor characteristics such as age, sex, and overall health status, the generalizability of our findings remains to be established. Future studies should validate these results using samples obtained from multiple donors with diverse demographic and clinical backgrounds to determine the reproducibility and robustness of the observed therapeutic effects. Finally, although several co-authors have disclosed professional relationships with the company developing BMAX™, the in vivo experiments were conducted at an independent academic institution, treatments were injected by a surgeon, HN, with no competing interests, and all behavioral, histological, and micro-computed tomography assessments were performed by investigators blinded to treatment allocation. Nevertheless, independent external validation will be important to further confirm the reproducibility of these findings. Despite these limitations, the study consistently demonstrated superior functional improvement and cartilage preservation with ECM-retaining micronized bone marrow compared with conventional BMAC under the conditions tested. These findings provide a rationale for future studies to optimize treatment protocols by evaluating multiple human donors, investigating dose–response relationships and repeated injection regimens, monitoring hematocrit and other product characteristics that may influence therapeutic efficacy, and assessing the long-term immunological safety and durability of treatment effects while further elucidating the underlying mechanisms of action. Finally, only one donor BMAX/BMAC was used for in vivo studies due to consideration of reduction in animal use and cost.

Taken together, the present findings support the concept that preservation of the native bone marrow microenvironment plays a critical role in optimizing the therapeutic performance of MSC-based interventions. By retaining ECM components and minimizing disruption of stromal architecture, micronized bone marrow demonstrated enhanced anti-inflammatory effects, improved movement-evoked pain, and superior cartilage preservation/regeneration compared with conventional BMAC in a preclinical model of post-traumatic osteoarthritis. These results reinforce emerging evidence that MSC efficacy is not solely dependent on cell number, but rather on the integrity of cell–matrix interactions that regulate MSC survival, plasticity, and paracrine signaling. Approaches that maintain the native stem cell niche may therefore represent an important advance in the development of the next generation of orthobiologic therapies.

## 5. Conclusions

This study demonstrates that retention of native extracellular matrix within a mechanically processed, micronized bone marrow preparation enhances the therapeutic potential of bone marrow-derived cell therapy in a preclinical OA model. Compared with conventional BMAC, ECM-retaining micronized bone marrow was associated with improved movement-evoked pain and greater regeneration of damaged articular cartilage, while both treatments showed comparable effects on subchondral bone architecture by micro-CT. These findings suggest that preservation of the native ECM and stem cell niche may enhance cartilage-regenerative effects and support the development of ECM-conscious strategies for future cell-based therapies for OA.

## Figures and Tables

**Table 1:** Von Frey pain threshold test results across different time points (mean ± SD, *n*).

Time Point	Control	BMAC	BMAX
Baseline	42.33 ± 14.98 (*n* = 6)	40.68 ± 9.12 (*n* = 6)	41.15 ± 10.27 (*n* = 5)
4 week	19.32 ± 4.65 (*n* = 6)	20.3 ± 5.55 (*n* = 6)	19.15 ± 2.99 (*n* = 5)
8 week	17 ± 6.72 (*n* = 6)	35.46 ± 8.9 (*n* = 6)	35.78 ± 13.35 (*n* = 5)
12 week	17.61 ± 5.75 (*n* = 6)	27.87 ± 7.15 (*n* = 6)	41.00 ± 13.30 (*n* = 5)

**Table 2:** Knee bend score results at different time points (Mean ± SD, *n*).

Time Point	Control	BMAC	BMAX
Baseline	0.33 ± 0.408 (*n* = 6)	0.25 ± 0.27 (*n* = 6)	0.2 ± 0.27 (*n* = 5)
4 week	6.67 ± 1.89 (*n* = 6)	7.17 ± 1.94 (*n* = 6)	7 ± 1.87 (*n* = 5)
8 week	5.67 ± 1.72 (*n* = 6)	3.08 ± 2.01 (*n* = 6)	0.8 ± 0.67 (*n* = 5)
12 week	6.5 ± 2.14 (*n* = 6)	3.17 ± 1.13 (*n* = 6)	1 ± 0.5 (*n* = 5)

**Table 3:** Knee diameter at different time points (Mean ± SD, *n*).

	Control	BMAC	BMAX
8 week	0.86 ± 0.12 (*n* = 6)	0.97 ± 0.46 (*n* = 6)	0.456 ± 0.12 (*n* = 5)
12 week	0.99 ± 0.11 (*n* = 5)	0.786 ± 0.11 (*n* = 5)	0.56 ± 0.20 (*n* = 5)

**Table 4:** OARSI histology score at 4 weeks after cell injections (Mean ± SD, *n*).

	Control	BMAC	BMAX
Femoral condyle	6.67 ± 8.14 (*n* = 3)	2.000 ± 2.19 (*n* = 6)	3.300 ± 3.31 (*n* = 5)
Tibial plateau	7.000 ± 5.57 (*n* = 3)	3.667 ± 4.59 (*n* = 6)	3.900 ± 4.59 (*n* = 5)

**Table 5:** OARSI histology score at 8 weeks after cell injections (Mean ± SD, *n*).

	Control	BMAC	BMAX
Femoral condyle	11.33 ± 4.04 (*n* = 3)	5.600 ± 3.647 (*n* = 5)	1.000 ± 0.7071 (*n* = 5)
Tibial plateau	17.33 ± 4.62 (*n* = 3)	5.000 ± 2.58 (*n* = 4)	2.700 ± 2.39 (*n* = 5)

## Data Availability

The original contributions presented in this study are included in the article/Supplementary Materials. Further inquiries can be directed to the corresponding authors.

## Supplementary Materials

The following supporting information can be downloaded at: https://www.mdpi.com/article/10.3390/cells15141249/s1, Figure S1: Histogram and dot plot gating criteria for flow cytometry. The cell population that were negative in the PE-channel for the WBC antibody cocktail (CD34^−^, CD45^−^, CD11b^−^, CD19^−^, HLA^−^DR^−^) were used as the starting criteria and backgated in a serial fashion for CD73^+^ (WBC^−^, CD73^+^), to CD105^+^ (WBC^−^, CD73^+^, CD105^+^) to CD90^+^ (WBC^−^, CD73^+^, CD105^+^, CD90^+^). Similarly, the appropriate antibody isotypes were used to set initial gates for the CD45 and CD146 panel. The CD146^+^ population in the respective channel was backgated to the CD45^−^ population 207 to determine the percent of CD45^−^/CD146^+^ cells.

## Abbreviations

The following abbreviations are used in this manuscript:

BMAC	Bone marrow aspirate concentrate
BMAX™	Micronized bone marrow preparation retaining extracellular matrix
BM	Bone marrow
BC	Bone core
BV/TV	Bone volume/total volume
CFU-F	Colony-forming unit–fibroblast
COL2	Type II collagen
DMM	Destabilization of the medial meniscus
ECM	Extracellular matrix
GAG	Glycosaminoglycan
HSC	Hematopoietic stem cell
IL	Interleukin
IRAP	Interleukin-1 receptor antagonist
IACUC	Institutional Animal Care and Use Committee
ISCT	International Society for Cell & Gene Therapy
MSC	Mesenchymal stromal/stem cell
OA	Osteoarthritis
OARSI	Osteoarthritis Research Society International
PBS	Phosphate-buffered saline
PTOA	Post-traumatic osteoarthritis
scRNA-seq	Single-cell RNA sequencing
Tb.N	Trabecular number
Tb.Sp	Trabecular separation
Tb.Th	Trabecular thickness
TNC	Total nucleated cells
TNF-α	Tumor necrosis factor alpha
