# Supplementary material for: Preclinical Evaluation of Human Donor-Derived Micronized Bone Marrow Stroma/Parenchyma Versus Bone Marrow Aspirate Concentrate in a Rat Model of Post-Traumatic Knee Osteoarthritis
Source: Cells. 2026 Jul 10;15(14):1249. doi: 10.3390/cells15141249 (PMC13407337; doi:10.3390/cells15141249)
Supplement: Supplementary file 1 [file cells-15-01249-s001.zip › cells-4399340-supplementary.pptx]

## Slide 1
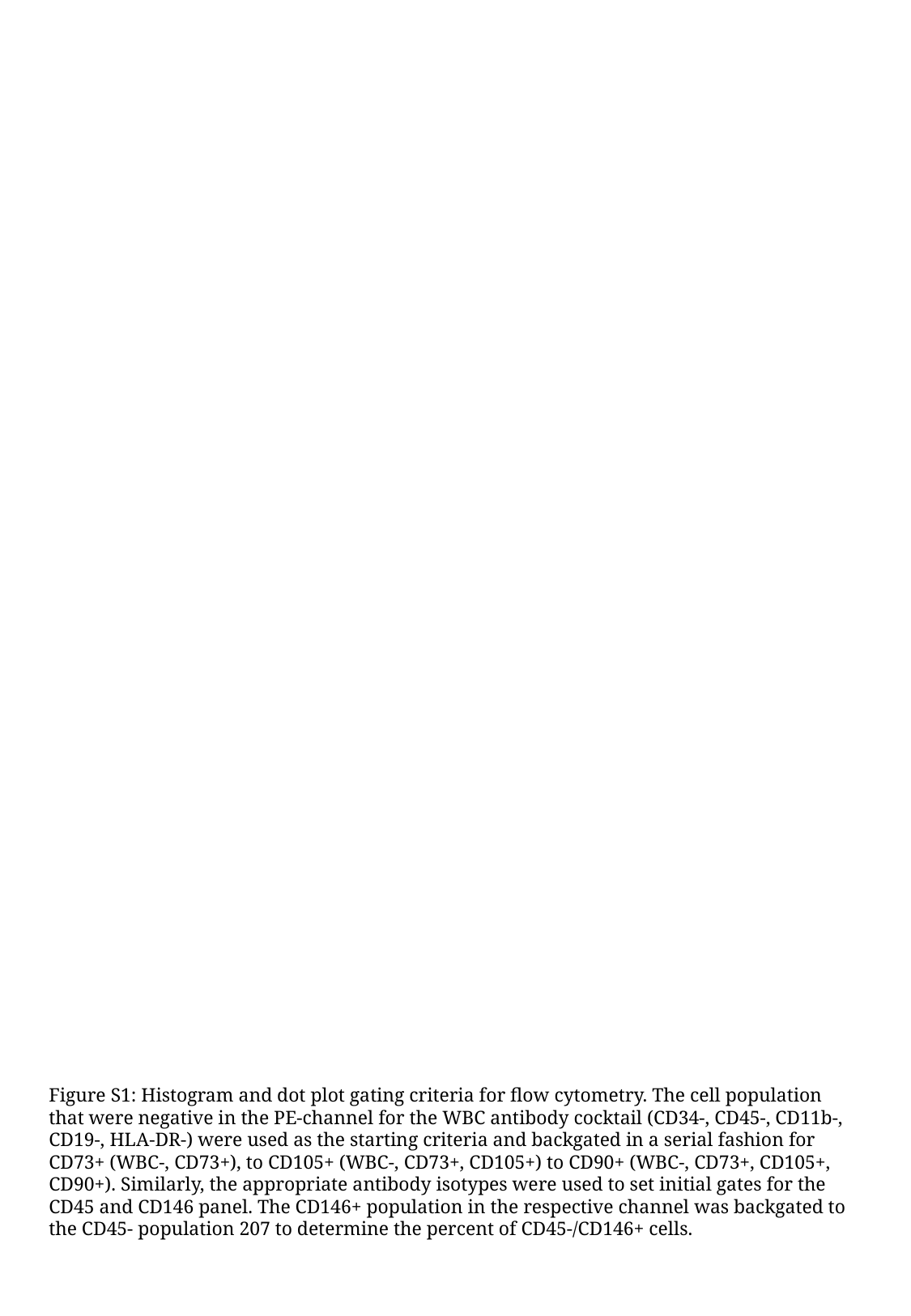

Figure S1: Histogram and dot plot gating criteria for flow cytometry. The cell population that were negative in the PE-channel for the WBC antibody cocktail (CD34-, CD45-, CD11b-, CD19-, HLA-DR-) were used as the starting criteria and backgated in a serial fashion for CD73+ (WBC-, CD73+), to CD105+ (WBC-, CD73+, CD105+) to CD90+ (WBC-, CD73+, CD105+, CD90+). Similarly, the appropriate antibody isotypes were used to set initial gates for the CD45 and CD146 panel. The CD146+ population in the respective channel was backgated to the CD45- population 207 to determine the percent of CD45-/CD146+ cells.
